# Analysis of microRNA expression in peripheral blood monocytes of three Traditional Chinese Medicine (TCM) syndrome types in psoriasis patients

**DOI:** 10.1186/s13020-020-00308-y

**Published:** 2020-05-01

**Authors:** Yue Lu, Yao Qi, Yuhong Yan, Danni Yao, Hao Deng, Jingwen Deng, Shuyan Ye, Haiming Chen, Qubo Chen, Hengjun Gao, Ling Han, Chuanjian Lu

**Affiliations:** 1 Guangdong Provincial Hospital of Chinese Medicine, Guangzhou, 510006 Guangdong People’s Republic of China; 2 Guangdong Academy of Traditional Chinese Medicine, Guangzhou, 510006 Guangdong People’s Republic of China; 3 The Second Clinical Medical College of Guangzhou University of Chinese Medicine, Guangzhou, 510006 Guangdong People’s Republic of China; 4 Guangdong Provincial Key Laboratory of Clinical Research on Traditional Chinese Medicine Syndrome, Guangzhou, 510006 Guangdong People’s Republic of China; 5 Shanghai Molecular Medicine Engineering Technology Research Center, Shanghai, 201203 China; 6 Shanghai National Engineering Research Center of Biochip, Shanghai, 201203 China

**Keywords:** miRNA chip, miRNA expression, Psoriasis, TCM syndrome type

## Abstract

**Background:**

To analyze the expression of miRNA (microRNA) in peripheral blood mononuclear cells in patients with *Psoriasis vulgaris* with different TCM syndromes by miRNA chip. It further revealed the micromaterial basis of different syndrome types of psoriasis at the miRNA level.

**Methods:**

Peripheral blood monocytes were collected and prepared from 30 patients with *Psoriasis vulgaris* (including 9 patients of blood heat syndrome, 8 patients of blood stasis syndrome, and 13 patients of blood dry syndrome) and 9 healthy controls. The miRNA expression profile of peripheral blood monocytes was detected by Agilent Hum miRNA chip.

**Results:**

Compared to the healthy control group, 156 upregulated and 242 downregulated miRNAs were detected in all psoriasis patients. Compared to the healthy control group, 40 miRNAs were upregulated and 44 were downregulated in the blood heat syndrome group. Furthermore, there were 49 upregulated miRNAs and 44 downregulated miRNAs in the dry syndrome group as compared to the healthy control group. Also, 67 miRNAs were upregulated and 154 miRNAs were downregulated in the blood stasis syndrome group as compared to the healthy control group.

**Conclusions:**

There are common different miRNAs and pathways, as well as specific miRNAs between the psoriasis and the healthy control groups.

*Trial registration* ChiCTR-TRC-14005185 registered on August 8, 2014.

## Background

Psoriasis is a chronic and recurrent skin inflammatory disease. In 2016, the World Health Organization (WHO) reported that the global prevalence of psoriasis ranged from 0.09 to 11.43% [1], and the incidence of psoriasis in China has increased from 0.12 to 1.49% in the past 25 years. Psoriasis has become a common disease endangering public health. Currently, there are defects in the treatment of psoriasis, such as a short remission period; also, the disease is easy to relapse or tolerate. Moreover, most drugs have considerable side effects. Traditional Chinese Medicine (TCM) treatment of psoriasis has the advantages of a long remission period, stable condition, not easy to rebound, and less toxicity and side effects. In recent years, it has attracted the attention of the medical community worldwide. Traditional Chinese medicine (TCM) has a unique theoretical system in the treatment of psoriasis and has accumulated abundant clinical experience for a prolonged period. TCM syndrome is the core of clinical TCM and vital for the modernization of TCM. Our research group analyzed the distribution of *Psoriasis vulgaris*-related literature from 1979 to 2010, and found that among all the syndromes of the disease, the top three were blood heat syndrome (32.86%), blood dry syndrome (23.56%), and blood stasis syndrome (19.43%), accounting for a total of 75.85% [2]. Moreover, blood stasis syndrome is related to a stable period, while the regression period is related to the blood dry syndrome [3].

Precision medicine is based on individualized medicine; it uses genomics, proteomics, and data analysis to achieve personalized and precise prevention and treatment of the diseases [4]. The connotation coincides with the doctrine “treatment based on syndrome differentiation” in traditional Chinese medicine. It can summarize the classification of TCM syndrome differentiation at the microscopic and molecular level based on the combination of genomics and proteomics to achieve accurate diagnosis and treatment as well as improve the therapeutic effect.

No correlation has been established between TCM classification of psoriasis and miRNA omics. In this study, we analyzed the differences of miRNA expression in common TCM types of psoriasis (blood heat syndrome, blood dry syndrome, and blood stasis syndrome) by miRNA microarray method and revealed the microscopic variations of the different syndrome types of psoriasis at the miRNA level.

## Materials and methods

### Recruitment and characteristics of patients

All patients were from the Dermatology Clinic and Ward of Guangdong Hospital of Traditional Chinese Medicine. This study was approved by the ethics committee of our hospital, and all patients and healthy individuals signed the informed consent. *Psoriasis vulgaris* group consisted of 30 patients, including 9 patients of blood heat syndrome, 8 patients of blood stasis syndrome, and 13 patients of the dry syndrome. The age of the patients was from 19 to 60 years, the duration of the disease was 1–10 years, the diagnostic standard was consistent with *Psoriasis vulgaris* [5], and the diagnosis of TCM syndrome was consistent with the diagnosis of *bai bi* syndrome [6]. The healthy control group comprised of 9 healthy patients as assessed in an outpatient physical examination and matched with the psoriasis group in gender and age. All the human studies were in accordance with the Helsinki declaration and Chinese relevant clinical trial research standards and regulations. All subjects included in this study were introduced to the purpose and treatment of this study by the researcher before specimen collection.

### Exclusion criteria

Pregnant or lactating women; patients administered steroids and/or retinoids or biological agents in the last six months; patients who used steroids or retinoids within 1 month; patients with primary diseases such as hyperthyroidism, cardiovascular, cerebrovascular, liver, kidney and hematopoietic system, as well as those on long-term medication; a variety of Chinese medicine syndrome type inclusion of patients.

### Instruments and reagents

TRI Reagent (catalogue no. T9424; Sigma, Germany), miRNeasy Micro Kit (cat alogue no. T9424 217084; Qiagen, Germany), RNase-Free DNase Set (cat alogue no. 79254; Qiagen), Tiangen miRcute miRNA cDNA First-Strand Synthesis Kit (cat alogue no. KR201; Tiangen, Beijing, China), SYBR Green PCR kit (cat alogue no. Fp411-02; Tiangen, Beijing, China), Agilent Bioanalyzer 2100 analyzer (Agilent Technologies, Santa Clara, CA, USA); Scanner G2505C (Agilent Technologies), feature extraction (version 10.7.1.1, Agilent Technologies), data processing software Genespring (version 13.1, Agilent Technologies), and Affymetrix Agilent Human miRNA, Release 21.0 (8 × 60K, Design ID: 070156) chip were utilized for this study.

### Sample collection and processing

A volume of 10 mL elbow vein blood was collected in an anticoagulant tube containing heparin, and peripheral blood monocytes (PBMCs) were separated according to the manufacturer’s instructions and stored at − 80 °C.

### Extraction and quality control of RNA

Total RNA was extracted using TRI reagent according to the manufacturer’s protocol and purified using the miRNeasy Micro Kit and RNase-Free DNase Set. During each RNA extraction experiment, a control total RNA was utilized as positive quality control and RNase-free water as negative quality control. After quality control (QA) evaluation, the integrity of the purified total RNA was detected by Agilent Bioanalyzer 2100 (Agilent technologies).

### Agilent miRNA microarray experiment

The chip used in this study was Agilent Human miRNA, Release 21.0 (8 × 60K, Design ID: 070156), and a total of 39 samples were on-chip and analyzed. Total RNA was quantified by NanoDrop ND-2000 (Thermo Scientific, MA, USA) and detected by Agilent Bioanalyzer 2100. After passing the RNA quality inspection, the samples were labeled, hybridized, and eluted according to the standard chip procedure. First, total RNA was dephosphorylated and denatured, followed by labeling with cyanine-3-CTP (Cy3). The labeled RNA was purified and hybridized with the chip. After elution, the original image was scanned by Agilent Scanner G2505C.

Feature Extraction software was used to extract the original data from the original image, and Genespring software was utilized for standardization and subsequent processing. The standardized data were filtered, and at least one set of 100% probes labeled “Detected” from each set of samples used for comparison was stored for subsequent analysis. P-values and multiple change values of T-test were used to screen differential miRNAs, and the screening criteria were upregulated or downregulated multiple change value ≥ 2.0 and P ≤ 0.05. Then, three databases (TargetScan, microRNA.org, PITA) were used to jointly predict the target genes of different miRNAs, followed by GO and KEGG enrichment analysis of the target genes to determine the biological functions or pathways mainly affected by different miRNAs. Finally, unsupervised hierarchical clustering was carried out for different miRNAs, and the expression patterns of different miRNAs in different samples were displayed in the form of a heatmap.

### Validation by quantitative real-time polymerase chain reaction (qRT-PCR)

The isolated RNA from the PBMC samples from different groups was reverse transcribed directly into cDNA using a SuperScript IV Reverse Transcriptase (Thermo Fisher, Dalian, China) according to the manufacturer’s instructions. PCR amplification conditions were as follows: initial denaturation at 95 °C for 15 min, followed by 40 cycles of denaturation at 94 °C for 20 s and annealing at 60 °C for 34 s. The expression of microRNAs was examined using qRT-PCR by a SYBR Green PCR Kit.

### Statistical analysis

Statistical analyses were performed using SPSS software 19.0 and represented using GraphPad Prism 5.0 (La Jolla, CA, USA). The results are expressed as means ± standard errors of the means. Student’s t-test was used for the statistical analysis. P-values < 0.05 were considered significant.

## Results

### Quality control of RNA chip data is satisfactory

**Fig. 1:**
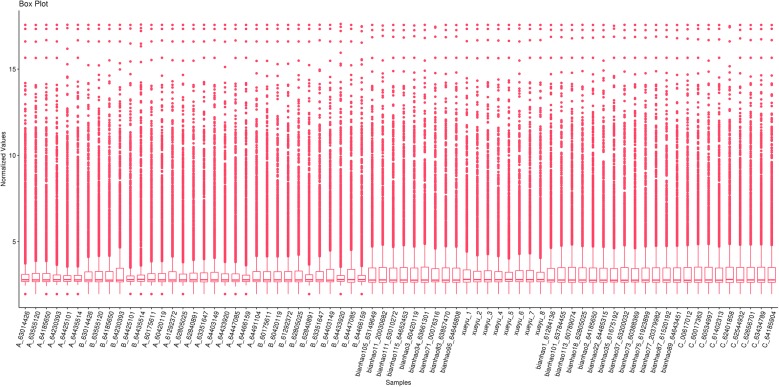
Quality control of RNA chip data is satisfactory

Combined with the clinical sample information, we selected 39 samples (9 samples of hematic psoriasis, 13 samples of dry psoriasis, 8 samples of blood stasis psoriasis, and 9 samples of the control group) to perform Agilent miRNA microarray analysis. The quality control of the original result data was satisfactory (Fig. 1).

### Analysis of miRNA microarray results of blood heat syndrome and control groups

**Fig. 2:**
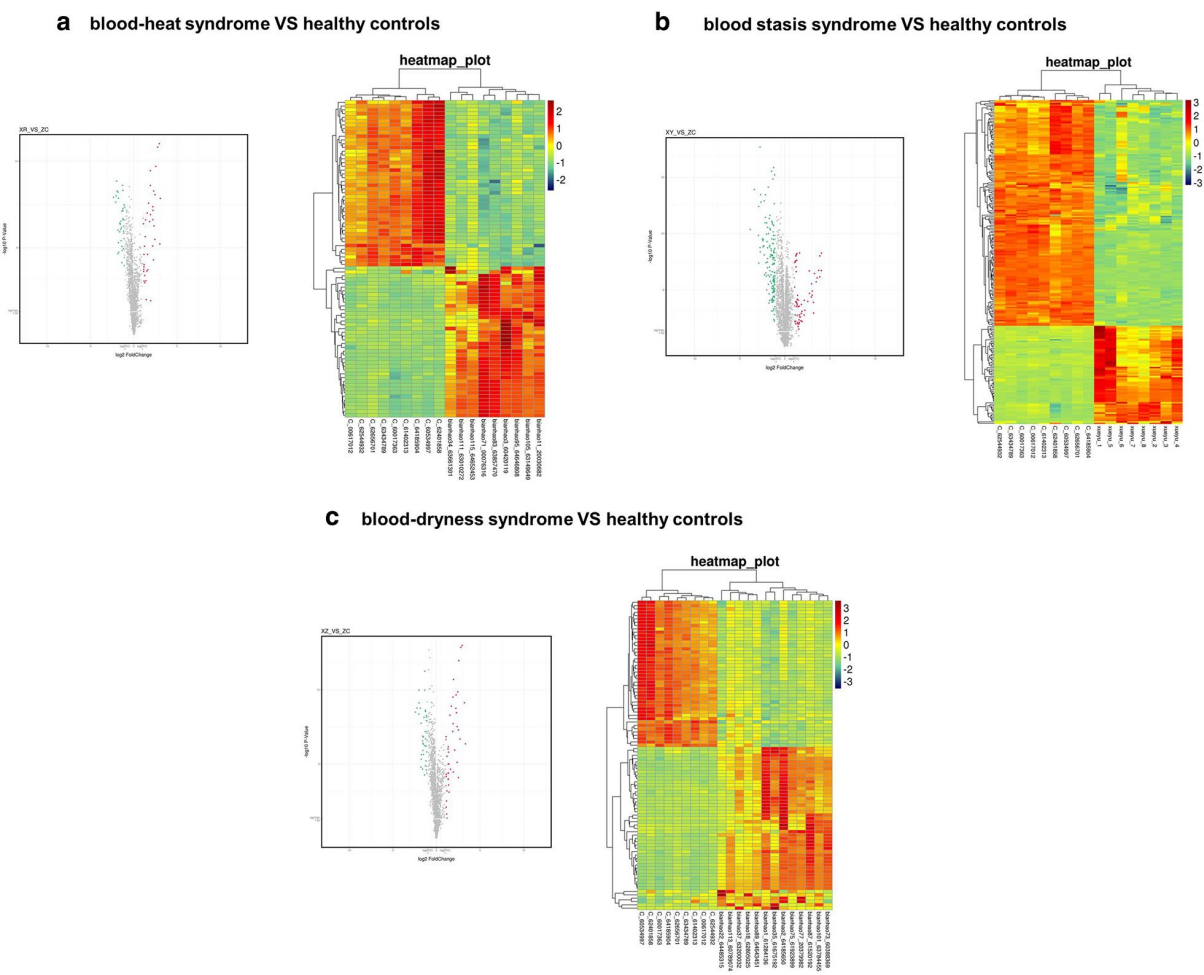
Volcanic map (left panel) and heat map (right) show the distribution of miRNAs in three different syndromes

**Fig. 3:**
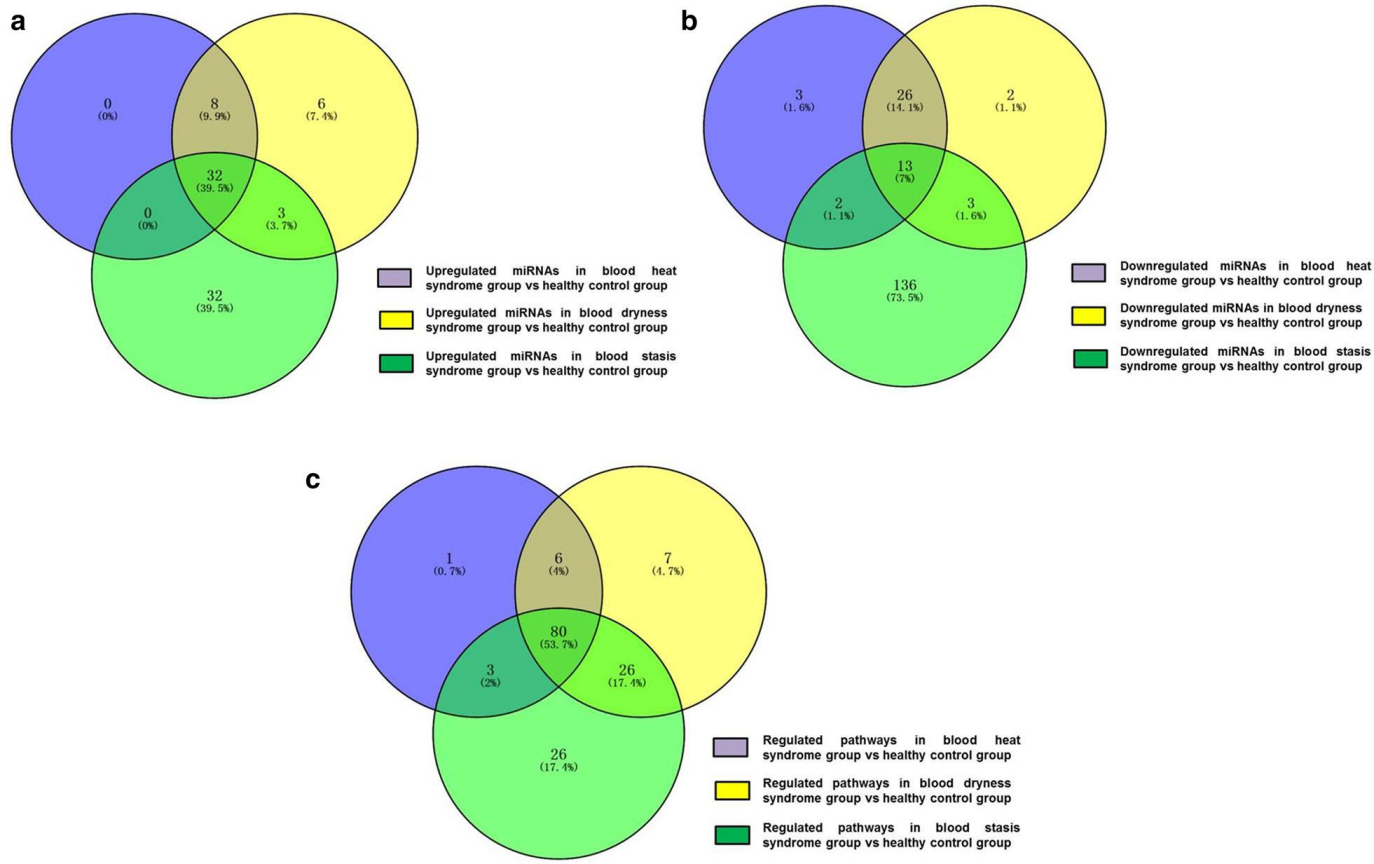
Wayne analysis of differential miRNAs and pathways between three syndromes of psoriasis and healthy control groups

Next, we conducted a preliminary analysis of miRNA microarray results from the blood heat syndrome and the control groups, and miRNAs with fold-change > 2 and P-value < 0.05 were screened out. Among these differential miRNAs, 40 were upregulated and 44 were downregulated. The Volcano Map was used to reflect the differences in the expression of miRNAs between the blood heat syndrome and the control groups (Fig. 2). The heatmap displayed the results of unsupervised hierarchical clustering of miRNAs differences between both groups (Fig. 2). In addition, Venn analysis of the differentially expressed genes compared the miRNA microarray data from both groups (Fig. 3).

**Fig. 4:**
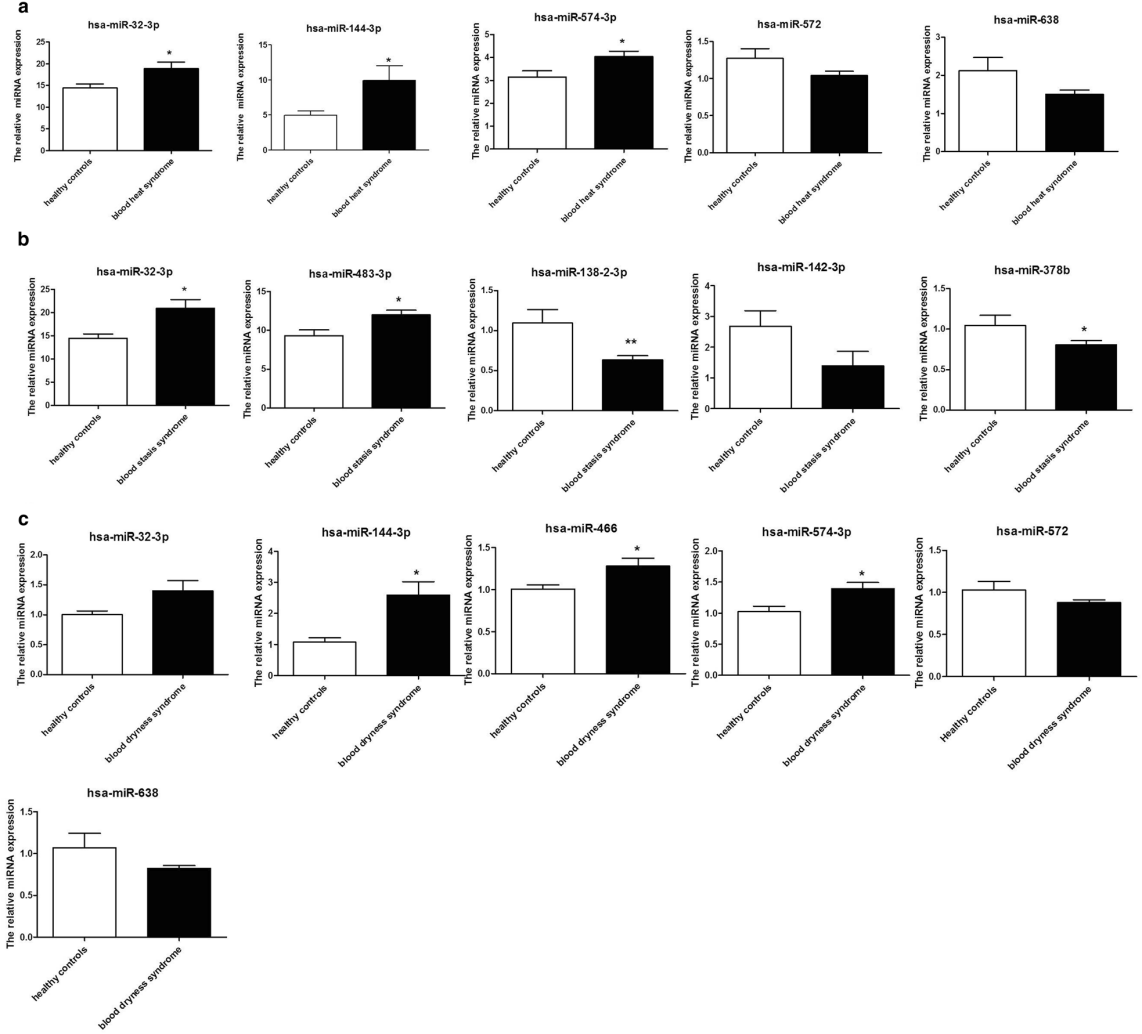
Differential miRNAs validated by qRT-PCR (*P < 0.05, **P < 0.01 in qRT-PCR verification while comparing the data of different syndrome groups to the healthy control group)

According to the results of the miRNA microarray, the first three upregulated miRNAs were miR-32-3p, miR-144-3p, and -miR-574-3p, and the first two downregulated miRNAs were miR-638 and miR-572. The verification results of qRT-PCR were consistent with those of miRNA microarray (Fig. 4).

### Analysis of miRNA microarray results of psoriasis blood dry syndrome and control groups

Herein, we conducted a preliminary analysis of miRNA microarray results in the blood dry syndrome and the control groups and miRNAs with fold-change > 2 and P-value < 0.05 screened out. Among these differential miRNAs, 49 were upregulated and 44 were downregulated. The Volcano Map reflected the differences in the expression of miRNAs between the blood dry syndrome and the control groups (Fig. 2), while the Heatmap showed the results of unsupervised hierarchical clustering of miRNA differences between both control group (Fig. 2). We also performed a Venn analysis of the differentially expressed genes to compare the miRNA microarray data in the blood dry syndrome and control groups (Fig. 3).

According to the results of miRNA microarrays, the first four upregulated miRNAs were miR-144-3p,miR-32-3p, miR-574-3p, and miR-466, and the first two down-regulated miRNAs were miR-638 and miR-572, and the verification results of qRT-PCR were consistent with the results of miRNA microarray (Fig. 4).

### Analysis of miRNA microarray results of psoriasis blood stasis syndrome and control groups

A preliminary analysis of miRNA microarray results was conducted in the blood stasis syndrome and the control groups, and miRNAs with fold-change > 2 and P-value < 0.05 were screened out. Among these differential miRNAs, 67 were upregulated and 154 were downregulated. The Volcano Map was used to reflect the differences in the expression of miRNAs between the blood stasis syndrome and the control groups (Fig. 2), and Heatmap showed the results of unsupervised hierarchical clustering of miRNAs differences between both groups (Fig. 2). Next, a Venn analysis of the differentially expressed genes was used to compare the miRNA microarray data from both group (Fig. 3).

According to the results of miRNA microarray, the first two upregulated miRNAs were miR-32-3p and miR-483-3p, and the first three downregulated miRNAs were miR-138-2-3p, miR-378b, and miR-142-3p; also, the verification results of qRT-PCR were consistent with those of miRNA microarray (Fig. 4).

### miRNA-enrichment pathways and of the three psoriasis syndromes were analyzed from the control group

After the target gene prediction of the different miRNAs in the control group, we used three databases (TargetScan, microRNA.org, and PITA) to enrich the pathways for the three psoriasis syndromes. Venn analysis was performed on the pathways of the three syndromes (Fig. 3).

As seen from Table 1, the unique differential pathway in the psoriatic blood heat syndrome group was pancreatic secretion, while that in the psoriatic blood dry syndrome group included Shigellosis, bacterial invasion of epithelial cells, endocrine, and other factor-regulated calcium reabsorption and Th17 cell differentiation. The unique differential pathway in the psoriatic blood stasis syndrome group included human T-cell leukemia virus 1 infection, calcium signaling pathway, and ECM-receptor interaction.

**Table 1:** Common and specific regulated pathways in different syndromes

The common pathways between the three syndromes (1)	The common pathways between the three syndromes (2)	Specific pathways in blood heat syndrome	Specific pathways in blood dryness syndrome	Specific pathways in blood stasis syndrome
Signaling pathways regulating pluripotency of stem cells	Thyroid hormone signaling pathway	Pancreatic secretion	Shigellosis	Human T-cell leukemia virus 1 infection
Proteoglycans in cancer	Glutamatergic synapse		Bacterial invasion of epithelial cells	Calcium signaling pathway
Breast cancer	Arrhythmogenic right ventricular cardiomyopathy (ARVC)		Endocrine and other factor-regulated calcium reabsorption	ECM-receptor interaction
Transcriptional misregulation in cancer	Hedgehog signaling pathway		Th17 cell differentiation	Cell cycle
Gastric cancer	Circadian entrainment		Insulin secretion	Inositol phosphate metabolism
Hepatocellular carcinoma	Non-small cell lung cancer		Glucagon signaling pathway	Glycosaminoglycan biosynthesis—heparan sulfate/heparin
Parathyroid hormone synthesis, secretion and action	Long-term depression		Epithelial cell signaling in Helicobacter pylori infection	Type II diabetes mellitus
Wnt signaling pathway	Neurotrophin signaling pathway			Measles
Focal adhesion	Phospholipase D signaling pathway			Mitophagy—animal
EGFR tyrosine kinase inhibitor resistance	Human papillomavirus infection			Amyotrophic lateral sclerosis (ALS)
Axon guidance	Renal cell carcinoma			Hippo signaling pathway—multiple species
Rap1 signaling pathway	Endometrial cancer			Protein digestion and absorption
Ras signaling pathway	mRNA surveillance pathway			Osteoclast differentiation
MAPK signaling pathway	Cholinergic synapse			NF-kappa B signaling pathway
TGF-beta signaling pathway	Choline metabolism in cancer			Nicotine addiction
Adherens junction	Adrenergic signaling in cardiomyocytes			Synaptic vesicle cycle
Glioma	GnRH signaling pathway			Apoptosis—multiple species
Melanoma	Yersinia infection			Apoptosis
FoxO signaling pathway	Long-term potentiation			Glycosaminoglycan biosynthesis—chondroitin sulfate /dermatan sulfate
Oxytocin signaling pathway	Insulin resistance			Platinum drug resistance
Prostate cancer	Ubiquitin mediated proteolysis			Vasopressin-regulated water reabsorption
cAMP signaling pathway	Platelet activation			SNARE interactions in vesicular transport
Hippo signaling pathway	Human cytomegalovirus infection			Cocaine addiction
Cushing syndrome	AMPK signaling pathway			JAK-STAT signaling pathway
Prolactin signaling pathway	Insulin signaling pathway			RNA transport
Circadian rhythm	Relaxin signaling pathway			Other types of O-glycan biosynthesis
Dopaminergic synapse	Cellular senescence			
Regulation of actin cytoskeleton	Fluid shear stress and atherosclerosis			
Gap junction	TNF signaling pathway			
Melanogenesis	Tight junction			
cGMP-PKG signaling pathway	Chagas disease (American trypanosomiasis)			
Endocrine resistance	Dilated cardiomyopathy (DCM)			
Apelin signaling pathway	Longevity regulating pathway			
Colorectal cancer	Pancreatic cancer			
Endocytosis	Longevity regulating pathway—multiple species			
Basal cell carcinoma	Sphingolipid signaling pathway			
Chronic myeloid leukemia	Central carbon metabolism in cancer			
PI3K-Akt signaling pathway	Aldosterone-regulated sodium reabsorption			
Morphine addiction	mTOR signaling pathway			
ErbB signaling pathway	Small cell lung cancer			

**Fig. 5:**
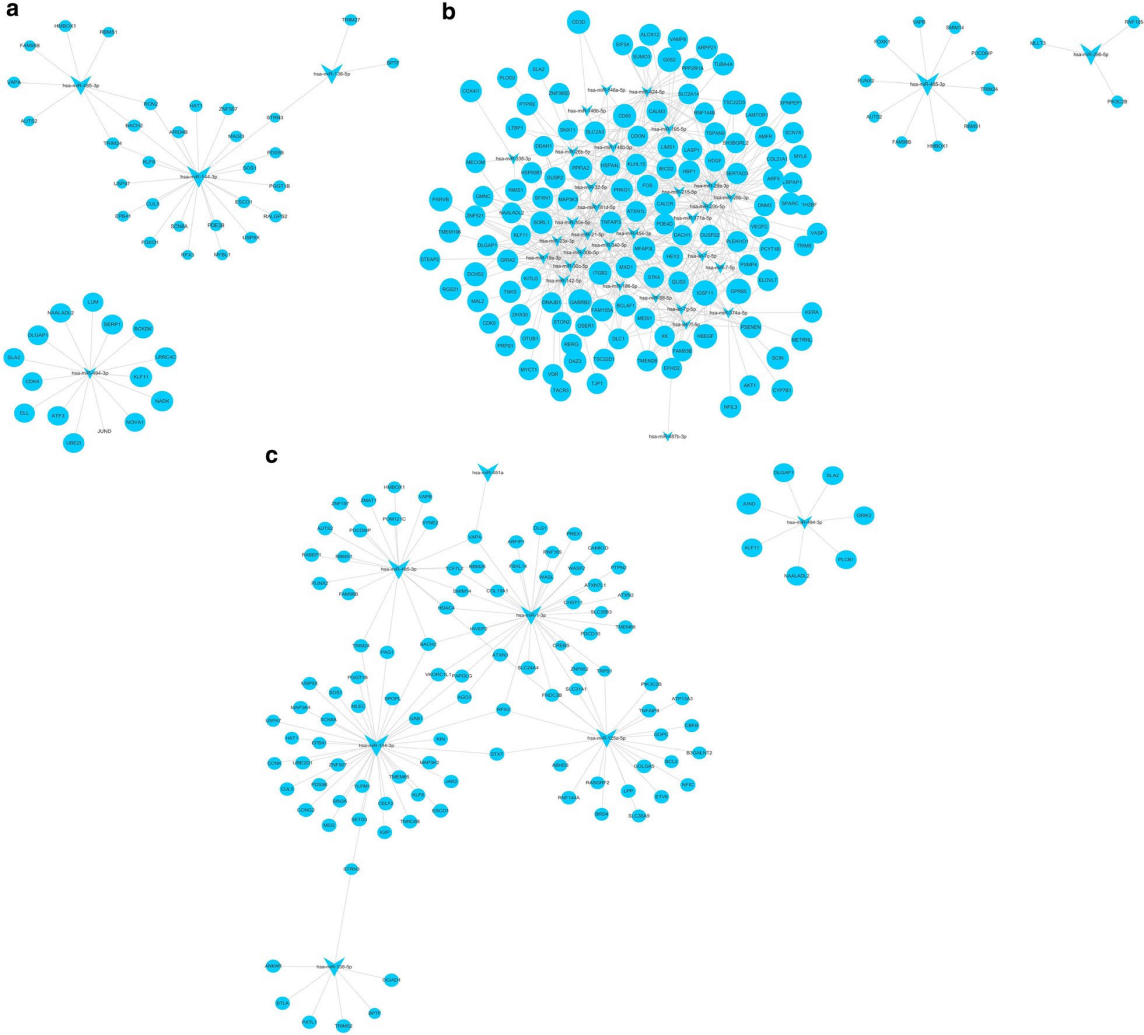
Correlation analysis of differentially expressed miRNAs and genes between three syndromes of psoriasis and healthy control groups. **a** Correlation analysis of differentially expressed miRNAs and genes between blood-heat syndrome and healthy control groups. **b** Correlation analysis of differentially expressed miRNAs and genes between blood-stasis syndrome and healthy control groups. **c** Correlation analysis of differentially expressed miRNAs and genes between blood-dryness syndrome and healthy control groups

Furthermore, the DIANA TOOLS online tool and its three databases (TarBase v7.0, microt-cds (v 5.0) and TargetScan) were used to correlate the differentially expressed miRNAs and genes (Fig. 5).

## Discussion

In this study, miRNA microarray was used to analyze the three most common TCM types of psoriasis at the level of miRNA expression. First, a preliminary analysis of miRNA microarray results was conducted and miRNAs with fold-change > 2 and P-value < 0.05 were screened out. Among these differential miRNAs, 40 were upregulated and 44 were downregulated in patients with blood heat, 49 were upregulated and 44 were downregulated in patients with blood dry, and 67 were upregulated and 154 were downregulated in patients with blood stasis as compared to the normal group. The comparison of the miRNA microarray data of the three psoriasis types of blood heat, blood stasis, and blood dry to that of the control group found that the three types of psoriasis had unique but different miRNAs as well as common miRNAs.

In the current analysis, compared to the control group, we found that the three syndromes of psoriasis had a common upregulated different miRNA: mir-32-3p. Reportedly, this miRNA is related to malignant pleural mesothelioma, acute ischemic stroke, chronic rhinosinusitis, and is also a potential biomarker [7–9]. The upregulation of miR-144-3p in both hematopoiesis and dry inhibits cell proliferation by negative regulation of the expression of Tie2 in pulmonary microvascular endothelial cells (PMVECs), and the overexpression of miR-144-3p can be used in the treatment of hepatopulmonary syndrome (HPS) [10]. In addition, miR-144-3p is significantly increased in the bone marrow and peripheral blood of patients with acute myeloid leukemia. miR-144-3p promotes the proliferation of HL-60 cells by inhibiting NRF2. Therefore, miR-144-3p can be considered as a non-invasive biological marker of leukemia [11]. The expression of miR-574-3p is upregulated in the syndrome of blood heat and dry, and the automatic regulation feedback loop of iASPP/p63 requires miR-574-3p to regulate homeostasis of the tunable and stratified epithelium [12]. In addition, miR-574-3p is also involved in the adipogenesis of the sebaceous gland [13]. The upregulation of miR-466 in dry syndrome is related to host immunity in the skin [14] and is involved in skin and mucosal HPV infection [15]. The upregulation of miR-483-3p in blood stasis syndrome is significantly increased in the plasma of pancreatic cancer patients [16], which suggests poor prognosis in pancreatic duct adenocarcinoma patients [17]. The miR-638 and miR-572 are significantly downregulated in basal cell carcinoma [18], suggesting their involvement in the pathogenesis of the disease. The downregulated miR-378b in blood stasis syndrome promotes keratinocyte differentiation by acting on NKX3.1 [19]. In addition, the downregulated miR-142-3p in blood stasis syndrome is a RANKL-dependent inducer of osteoclast death [20].

Next, we predicted the different miRNA target genes between the three syndromes and the control group and carried out KEGG enrichment. After Venn analysis on the pathway, we found that the three syndromes had a common as well as a specific pathway. The common different pathways of the three syndromes, signaling pathways regulating the pluripotency of stem cells, needed the activation of MAPK and PI3K signaling pathways that are closely related to the occurrence of psoriasis [21]. A PI3K-Akt signaling pathway is associated with the occurrence and development of psoriasis. A previous study reported that PI3K signaling regulates the proliferation of keratinocytes by activating Akt and other targets and inducing the downregulation of FOXO [22]. In addition, the PI3K-Akt-mTOR pathway induces keratinocyte proliferation and inflammatory cytokine secretion by inhibiting autophagy in keratinocytes. The inhibition of the PI3K-Akt-mTOR pathway alleviates the psoriasis symptoms [23–28]. The common different pathways of the three syndromes include the MAPK signaling pathway. The MAPK signaling pathway is a serine/threonine protein kinase, which mainly includes extracellular signal-regulated protein kinases (ERK), C-jun N-terminal kinases (JNK), and p38 MAPK. Some studies have shown that excessive activation of p38 MAPK leads to decreased function of the skin barrier in psoriasis as well as skin inflammation [29–32].

The common pathway of the three syndromes is axon guidance, which is involved in the occurrence and development of psoriasis. Reportedly, the axonal guide molecules, CD100 and Plexin-B2, are involved in the inflammatory process of psoriasis by activating NF-κB and NLRP3 inflammasome in keratinocytes [33].

The specific pathway of dry psoriasis includes Th17 cell differentiation. Genetic studies have reinforced that Th17 cells play a central role in psoriasis [34]. These cells are responsible for the production of the cytokine IL-17A. TGF- β with IL -6 or IL -21 causes initial CD4+ T cells to differentiate into the Th17 subgroup, and IL -23, which is the growth-stabilizing factor that affects the normal secretion process of IL-17. A large number of Th17 cells were infiltrated into the lesions of psoriasis. Lewis et al. [35] isolated and counted Th17 cells in the plaque tissues of patients, and found the proportion of CD4+ Th cells was 49–93%. Th17 cells exert an effect by secreting IL-17A, IL-17F, IL-26, and TNF in psoriasis, forming a feedforward inflammatory network with KCs, activating STAT-1 and NF-κB signaling pathway, and inducing keratinocytes (KCs) to secrete several pro-inflammatory factors. IL-17 and TNF can jointly stimulate the high expression of KCs CCL20 [36] to collect the activated CD11c + macrophage-derived dendritic cells (mDCs) and adjacent CCR6 + T17 cells and induce the differentiation of T cells in skin drainage lymph nodes towards Th17 and Tc17.

The human T-cell leukemia 1 infection pathway is abnormal in blood stasis psoriasis. A previous study showed that ustekinumab could successfully treat psoriasis with human -cell leukemia/type 1 lympho virus infection [37]. Further analysis found that osteoclast differentiation, a unique pathway, was abnormal in blood stasis psoriasis. Raimondo et al. demonstrated that skin inflammation in psoriasis promoted the differentiation of human monocytes into active osteoclasts and bone injury [38].

## Conclusions

In this study, we connected three common TCM syndromes of psoriasis with miRNAs and provided a reference for understanding the TCM syndromes of psoriasis. In the future study, we will explore the mechanism of the differentially expressed miRNAs from different TCM syndromes.

## Data Availability

The datasets used and/or analyzed during the current study would be available from the corresponding author on reasonable request.

## Abbreviations

TCM: Traditional Chinese Medicine
PBMCs: Peripheral blood mononuclear cells
qRT-PCR: Quantitative real-time polymerase chain reaction
miRNA: microRNA
PMVECs: Pulmonary microvascular endothelial cells
HPS: Hepatopulmonary syndrome
ERK: Extracellular signal-regulated protein kinases
JNK: C-jun N-terminal kinases
KCs: Keratinocytes
mDCs: Macrophage-derived dendritic cells
